# Diagnostic yield, safety, and impact of transbronchial lung biopsy in mechanically ventilated, critically ill patients: a retrospective study

**DOI:** 10.1186/s12890-020-01357-7

**Published:** 2021-01-07

**Authors:** Alessandro Ghiani, Claus Neurohr

**Affiliations:** 1 Department of Pulmonary and Respiratory Medicine, Schillerhoehe Lung Clinic (Robert-Bosch-Hospital GmbH, Stuttgart), Solitudestr. 18, 70839 Gerlingen, Germany; 2 German Center for Lung Research (DZL), Germany, Germany

**Keywords:** Transbronchial lung biopsy, Mechanical ventilation, Critical illness, Safety, Diagnostic yield, Organizing pneumonia

## Abstract

**Background:**

Pulmonary infiltrates of variable etiology are one of the main reasons for hypoxemic respiratory failure leading to invasive mechanical ventilation. If pulmonary infiltrates remain unexplained or progress despite treatment, the histopathological result of a lung biopsy could have significant impact on change in therapy. Surgical lung biopsy is the commonly used technique, but due to its considerable morbidity and mortality, less invasive bronchoscopic transbronchial lung biopsy (TBLB) may be a valuable alternative.

**Methods:**

Retrospective, monocentric, observational study in mechanically ventilated, critically ill patients, subjected to TBLB due to unexplained pulmonary infiltrates in the period January 2014 to July 2019. Patients’ medical records were reviewed to obtain data on baseline clinical characteristics, modality and adverse events (AE) of the TBLB, and impact of the histopathological results on treatment decisions. A multivariable binary logistic regression analysis was performed to identify predictors of AE and hospital mortality, and survival curves were generated using the Kaplan-Meier method.

**Results:**

Forty-two patients with in total 42 TBLB procedures after a median of 12 days of mechanical ventilation were analyzed, of which 16.7% were immunosuppressed, but there was no patient with prior lung transplantation. Diagnostic yield of TBLB was 88.1%, with AE occurring in 11.9% (most common pneumothorax and minor bleeding). 92.9% of the procedures were performed as a forceps biopsy, with organizing pneumonia (OP) as the most common histological diagnosis (54.8%). Variables independently associated with hospital mortality were age (odds ratio 1.070, 95%CI 1.006–1.138; *p* = 0.031) and the presence of OP (0.182, [0.036–0.926]; *p* = 0.040), the latter being confirmed in the survival analysis (log-rank p = 0.040). In contrast, a change in therapy based on histopathology alone occurred in 40.5%, and there was no evidence of improved survival in those patients.

**Conclusions:**

Transbronchial lung biopsy remains a valuable alternative to surgical lung biopsy in mechanically ventilated critically ill patients. However, the high diagnostic yield must be weighed against potential adverse events and limited consequence of the histopathological result regarding treatment decisions in such patients.

## Background

Intubation with mechanical ventilation is a life–saving procedure for patients with acute severe hypoxemic respiratory failure due to pulmonary infiltrates of variable etiology [1]. If infiltrates remain unexplained or progress despite therapy (e.g. antibiotics), it is almost impossible to derive a specific diagnosis based solely on clinical symptoms, radiological findings, and laboratory values. Empiric treatment of such patients tends to over–therapy, that may have potentially toxic side effects (e.g. for unnecessary application of broad-spectrum and long-term antimicrobial agents). This also generates high costs and rare and potentially reversible causes of pulmonary infiltrates remain undetected and untreated. In contrast, the histopathological result of a lung biopsy may provide important information on the underlying disease and could have significant impact on treatment decisions. Surgical lung biopsy (SLB) is the commonly used technique in such patients [2, 3], but hypoxemia may worsen dramatically with single–lung ventilation, and the procedure usually requires a transfer from the intensive care unit (ICU) to the operating theater. Alternatively, bronchoscopic, transbronchial lung biopsy (TBLB, by means of forceps biopsy or cryobiopsy) is available, which also can be performed at the bedside in the ICU in the event of mechanical ventilation [4–9].

The present study aims to assess the diagnostic yield, safety, and therapeutic consequences of transbronchial lung biopsy in a cohort of mechanically ventilated, critically ill patients.

## Methods

Exploratory, retrospective, monocentric, observational study on mechanically ventilated, critically ill patients, treated at the Schillerhoehe Lung Clinic (Gerlingen, Germany) from January 2014 to July 2019, and subjected to transbronchial lung biopsy due to unexplained pulmonary infiltrates. The study was approved by the local ethics committee, the need for informed consent was waived (Ethics Committee of the State Chamber of Physicians of Baden-Wuerttemberg, Germany, file number F–2019–096).

### Patient selection

Patients were identified using the 2019 Diagnosis Related Groups (DRG) codes for mechanical ventilation (DRG A06, A07, A09, A11, A13, E40, F43) and the modified International Classification of Procedures in Medicine (ICPS) code for the TBLB (OPS 1–430.2).

### Data collection

Data were collected from the hospitals’ electronic medical record and chart systems (PDMS Metavision ICU, iMDsoft, Tel Aviv, Israel; iMedOne, Telekom Healthcare Solutions, Bonn, Germany), and from the prospectively maintained records of the bronchoscopy database (ViewPoint 6, GE Healthcare GmbH, Chalfont St. Giles, Great Britain). These data included patient’s baseline characteristics on ICU admission, such as demographic data, leading cause for intubation, presence of acute respiratory distress syndrome (ARDS) defined by the Berlin criteria [10], and comorbidities, as well as modalities and adverse events (AE) of TBLB.

AE assessed were pneumothorax, minor and major bleeding, hemodynamic instability (defined as either a start or increase in dosage of vasopressors during the procedure), and death. Minor bleeding was defined as bleeding control by means of segmental wedging and/or topical administration of cold saline or adrenaline, whereas major bleeding required an additional hemostatic agent (e.g. oxidized regenerated cellulose [ORC] mesh), pulmonary isolation (using selective endobronchial intubation, a bronchus blocker or a double-lumen tube), bronchial artery embolization or surgery [11].

The histopathological results of TBLB were assessed for specific histological diagnoses. Furthermore, subgroups of patients depending on histological findings (e.g. patients with organizing pneumonia [OP], either cryptogenic [COP] or secondary to an underlying disease [SOP]) were separately analyzed. Changes in therapy based on the histopathological result (e.g. commencement of corticosteroid treatment or immunosuppression) were recorded. We defined responsiveness to corticosteroids as an increase in the ratio of partial pressure of oxygen to fraction of inspired oxygen (P/F ratio) of more than 100 mmHg within one week of therapy, as previously described [12, 13].

### Transbronchial lung biopsy

All bronchoscopies were carried out by an experienced interventional pulmonologist who was familiar with both the flexible and the rigid bronchoscopy technique. TBLB was performed either at the bedside in the ICU or in the bronchoscopy unit. Target lobes and lung segments were selected based on a current chest CT scan. TBLB was always performed unilaterally to avoid bilateral pneumothorax. The main criterion for exclusion was severe coagulopathy with thrombocytopenia < 50/nL, activated partial thromboplastin time (aPTT) > 50 s, an International Normalized Ratio (INR) > 1.5, and the presence of anticoagulants or antiaggregants (with the exception of acetylsalicylic acid [14]). Biopsies were always performed after broncho-alveolar lavage (BAL) and in different lung segments as for BAL. A therapeutic bronchoscope (BF–1 T180, Olympus Corporation, Tokyo, Japan) was introduced to the endotracheal tube or tracheal cannula through a special adapter (Smoothbore connector, Intersurgical, Sankt Augustin, Germany) to avoid air-leaks. All patients were deeply analgesized, using midazolam/propofol and sufentanil, and muscle relaxed (Cis-atracurium, 0.15 mg/kg). Fraction of inspired oxygen (FiO_2_) was set at 1.0 and ventilator settings were adjusted to counteract a drop in tidal volume in the pressure-controlled ventilation mode during bronchoscopy. TBLB in the ICU was performed at the bedside usually without fluoroscopic control. The number of biopsies was determined by the operator, with usually 4–6 biopsies per lobe obtained. For biopsy, either a 2 mm alligator biopsy forceps (2.0 mm fenestrated Swing Jaw, Olympus Corporation, Tokyo, Japan) or a 1.9 mm cryoprobe (Erbe Elektromedizin GmbH, Tübingen, Germany) was used. The decision for either the transbronchial forceps biopsy or cryobiopsy was at the discretion of the treating pulmonologist. Retrieved biopsy samples were immediately placed in formalin solution. Two hours after completion of the procedure, a chest X-ray was performed to rule out pneumothorax.

### Statistical analysis

Descriptive and frequency statistics were used to summarize patients’ demographics and baseline characteristics. Data are reported as mean/standard deviation for continuous variables and number/percentages for categorical variables. Differences in categorical variables between groups were analyzed using the Chi-square test or Fisher’s exact test, as appropriate. Continuous variables were subjected to Kolmogorov-Smirnov normality test for homogeneity of variance, and according to statistical distribution, Student’s *t*-test or Mann-Whitney *U*-test was used to examine differences in these parameters. We performed a binary logistic regression analysis (using forward selection) to derive variables independently associated with AE of the TBLB and hospital mortality. Survival curves were generated using the Kaplan-Meier method, compared by log-rank test. All statistical tests were two-tailed and statistical significance was considered for *p* < 0.05. All analyses were performed using MedCalc statistical software version 19.2.5 (MedCalc software Ltd., Ostend, Belgium).

## Results

Forty-two patients with in total 42 TBLB procedures were assessed, of which seven patients (16.7%) were immunosuppressed, but there was no patient with prior lung transplantation. Three patients were on Methotrexate due to rheumatoid arthritis and polymyalgia rheumatica, two patients on Cyclophosphamide pulse therapy due to interstitial lung disease (ILD) associated with hypersensitivity pneumonia and systemic sclerosis, one patient was on neoadjuvant chemotherapy with Cisplatin/Paclitaxel due to non-small cell lung cancer, and one patient received Tacrolimus due to focal segmental glomerulosclerosis. The most common clinical diagnosis leading to mechanical ventilation was pneumonia (52.4%), and 25 patients (59.5%) met the ARDS Berlin criteria (Table 1).

**Table 1:** Baseline demographics and clinical characteristics on ICU admission – comparison of patients with and without histologically confirmed organizing pneumonia

Variable	All patients (*n* = 42)	Patients with OP (*n* = 23)	Patients without OP (*n* = 14)	***P*** value^***a***^
**Clinical characteristics**				
Age (years)	60.5 (± 16.1)	66.0 (± 15.4)	63.4 (± 18.8)	0.876^*c*^
Gender (male)	26 (61.9)	13 (56.5)	9 (64.3)	0.645^*d*^
APACHE II (points)	20.2 (± 6.4)	19.6 (± 6.3)	19.9 (± 7.2)	0.887^*b*^
Albumin (g/dL)	2.2 (± 0.7)	2.3 (± 0.8)	2.0 (± 0.5)	0.082^*c*^
Vasopressors	28 (66.7)	16 (69.6)	9 (64.3)	0.054^*d*^
Hemodialysis	6 (14.3)	4 (17.4)	1 (7.1)	0.383^*d*^
Surgery prior to ARF	12 (28.6)	10 (43.5)	2 (14.3)	0.084^*e*^
Berlin ARDS criteria fulfilled*	25 (59.5)	15 (65.2)	9 (64.3)	0.955^*d*^
*Mild (P/F 200–300)*	2 (8.0)	2 (13.3)	0 (0.0)	–
*Moderate (P/F 100–200)*	19 (76.0)	11 (73.3)	7 (77.8)	–
*Severe (P/F < 100)*	4 (16.0)	2 (13.3)	2 (22.2)	–
ECLA	8 (19.0)	4 (17.4)	4 (28.6)	0.445^*e*^
**Cause of respiratory failure**				
Pneumonia	22 (52.4)	12 (52.2)	8 (57.1)	0.772^*d*^
AE–ILD	8 (19.0)	4 (17.4)	3 (21.4)	1.000^*e*^
Surgery	7 (16.7)	5 (21.7)	2 (14.3)	0.687^*e*^
Other	5 (11.9)	2 (8.7)	1 (7.1)	1.000^*e*^
**Comorbidities**				
CCI (points)	5.1 (± 2.5)	5.2 (± 2.1)	4.7 (± 3.1)	0.476^*c*^
Diabetes mellitus	10 (23.8)	5 (21.7)	4 (28.6)	0.705^*e*^
Chronic kidney disease	10 (23.8)	7 (30.4)	3 (21.4)	0.710^*e*^
Coronary artery disease	10 (23.8)	5 (21.7)	3 (21.4)	1.000^*e*^
Immunosuppression	7 (16.7)	4 (17.4)	3 (21.4)	1.000^*e*^

Table S1 describes the CT features of each patient in detail (see Additional file 1); all patients had bilateral infiltrates on chest CT scan at the time of the TBLB. Median time from intubation to TBLB was 12 days (range 4–98 days). A transbronchial forceps biopsy was performed in 92.9% of cases, 83.3% of the biopsies were performed in the ICU, and fluoroscopy was used in 11.9% of all procedures (and always for cryobiopsy). Transbronchial cryobiopsy in three patients (7.1%) was performed in the bronchoscopy unit using either a rigid bronchoscope (twice) or an endotracheal tube (once), without prophylactic balloon placement. Right lung biopsy was performed in 57.1 and 50% of all samples were taken from two lung lobes (most often from the right upper [RUL] and right lower lobe [RLL]). 42.9% of all biopsies were performed under chest drainage protection (Table 2).

**Table 2:** Modality of transbronchial lung biopsy

Modality	n (%)
**No. of procedures**	42
**Type of procedure**	
Forceps biopsy	39 (92.9)
Cryobiopsy	3 (7.1)
Fluoroscopy-guided biopsy	5 (11.9)
**Place of procedure**	
Intensive care unit	35 (83.3)
Bronchoscopy unit	7 (16.7)
**Airway access**	
Endotracheal tube	27 (64.3)
Tracheal cannula	13 (31.0)
Rigid bronchoscope	2 (4.8)
**Ventilator variables & respiratory indices**	
P/F ratio (mmHg)	192.2 (± 89.8)
FiO_2_	0.58 (± 0.22)
IPAP (cmH_2_O)	27.2 (± 4.8)
PEEP (cmH_2_O)	8.6 (± 2.9)
LTC_dyn_ (mL/cmH_2_O)	31.6 (± 15.4)
Mechanical power (J/min)	26.2 (± 9.3)
**Coagulation parameters**	
aPTT (sec)	39 (± 12)
INR	1.2 (± 0.2)
Platelet count (per μL)	263 (± 139)
**Anticoagulants/Antiaggregants**	
No. of patients	21 (50.0)
Thrombosis Prophylaxis	12 (28.6)
Thrombosis Prophylaxis & ASS	7 (16.7)
ASS	2 (4.8)

Continuous variables are presented as mean values (± standard deviation); categorical variables are presented as number or number (%). Thrombosis Prophylaxis refers to Enoxaparin (≤ 40 mg/day s.c.) or unfractionated heparin (≤ 15.000 U/day s.c)

*Abbreviations:* P/F ratio, ratio of partial pressure of oxygen to fraction of inspired oxygen, *FiO*_*2*_ fraction of inspired oxygen, *IPAP* inspiratory positive airway pressure, *PEEP* positive end-expiratory pressure, *LTC*_*dyn*_ dynamic lung-thorax compliance, *INR* International Normalized Ratio, *aPTT* activated partial thromboplastin time, *ASS* acetylsalicylic acid (100 mg/day)

The median effective number of specimens obtained (and ultimately analyzed by the pathologists) in 40 patients was 4 (range 2–9) and the median size of these specimens was 3 mm (range 2–7 mm); this information was missing in two patients (4.8%). Diagnostic yield of the TBLB was 88.1%, meaning that TBLB revealed a specific histological diagnosis in 37 patients, with OP as the most common one in the whole study population (54.8%) (see Additional file 1: Table S2) and in patients fulfilling the ARDS criteria (60.0%). Sixteen patients (38.1%) were clinically classified as SOP (9 patients with pneumonia as the leading cause of intubation, 6 postoperative patients, and 1 patient with ANCA-associated vasculitis) and seven patients (16.7%) as COP. A diagnosis of drug-induced lung injury (DILI) was made in four patients, of which three patients experienced Amiodarone-induced pulmonary toxicity, and another four patients showed diffuse alveolar damage (DAD). In five patients (11.9%), either there was no lung tissue in the biopsy sample or the histological pattern was not classifiable. No biopsy showed more than one histological diagnosis, and no patient underwent subsequent SLB. Bronchial lavage (BL) was performed in 41 patients (97.6%), and 21 patients (50.0%) were subjected to BAL. There was evidence of infection from BL in 11 patients (26.8%). The mean amount of aspirated liquid in the BAL was 33 mL (± 10 mL); with pure neutrophilia as the most frequent cell distribution in the whole population (61.9%) and in patients with OP (66.7%) (Table 3, see Additional file 1: Table S3).

**Table 3:** Results of transbronchial lung biopsy, bronchial lavage and BAL

Specimen	n (%)
**Transbronchial lung biopsy**	**42**
Organizing pneumonia	23 (54.8)
Diffuse alveolar damage	4 (9.5)
Drug-induced lung toxicity	4 (9.5)
Purulent bronchopneumonia	2 (4.8)
Acute exacerbation of ILD	1 (2.4)
Granulomatous disease (Tb)	1 (2.4)
Non-small cell lung cancer	1 (2.4)
Silicosis	1 (2.4)
**Bronchial lavage**	**41**
Cytomegalovirus (PCR)	3
*Pseudomonas aeruginosa* (culture)	3
Herpes simplex virus (PCR)	1
Aspergillus fumigatus (culture)	1
Klebsiella oxytoca (culture)	1
Stenotrophomonas maltophilia (culture)	1
*Enterococcus faecalis* (culture)	1
Pneumocystis jiroveci (PCR)	1
*Mycobacterium tuberculosis* (culture)	1
**Broncho-alveolar lavage (BAL)**	**21**
Neutrophilia	13 (61.9%)
Neutrophilia, Eosinophilia	3 (14.3%)
Neutrophilia, Eosinophilia, Lymphocytosis	2 (9.5%)
Neutrophilia, Lymphocytosis	1 (4.8%)
Lymphocytosis	1 (4.8%)
Normal cell distribution	1 (4.8%)

Normal cell distribution of BAL refers to ≥85% of alveolar macrophages, ≤ 15% lymphocytes, ≤ 3% neutrophils, and ≤ 1% eosinophils

*Abbreviations: OP* organizing pneumonia, *ILD* interstitial lung disease, *Tb* tuberculosis, *PCR* polymerase chain reaction

Eight adverse events were recorded in five patients (11.9%). Pneumothorax, occurring in three patients (7.1%) who all required a chest drainage, was amongst the most common AE. However, TBLB was performed with chest drainage protection in 18 patients, so that frequency increased to 12.5% when pneumothoraces were related to patients without such a protection. One pneumothorax occurred after cryobiopsy and the others occurred as a result of forceps biopsy, but no patient developed a persistent air leak. Biopsies leading to pneumothorax were performed in the RUL/RLL (twice) and in the RLL (once). Minor bleeding occurred in 7.1%, but there was no major bleeding event. One patient died as result of forceps biopsy with tension pneumothorax and persistent hemodynamic instability with shock despite immediate chest tube insertion (Table 4).

**Table 4:** Adverse events of transbronchial lung biopsy

Adverse events	n (%)
**No. of patients with AE**	5 (11.9)
**No. of AE**	8
**Pneumothorax**	
All patients	3 (7.1)
Patients without chest drainage	3 (12.5)
**Minor bleeding**	3 (7.1)
**Major bleeding**	0 (0.0)
**Hemodynamic instability**	1 (2.4)
**Death**	1 (2.4)

Categorical variables are presented as number (%)

*Abbreviations: AE* adverse event(s)

The histopathological results of the TBLB resulted in a change in therapy in 17 patients (40.5%). Corticosteroids were initiated in 15 patients (12 patients with OP, two patients with DILI, and one patient with DAD); one patient with pre-existing and acute exacerbated ILD was switched from cyclophosphamide pulse therapy to rituximab, and one patient with a histological diagnosis of DAD discontinued corticosteroid treatment.

In patients with corticosteroid induction a median cumulative dose of 600 mg [range 500–4000 mg] of prednisolone was administered within the first week. Responsiveness to corticosteroids with marked improvement in gas exchange (as defined above) could be observed in five patients (which all had histologically confirmed OP; median increase in P/F ratio by day seven of 127 mmHg [105–137 mmHg], compared to 15.5 mmHg [− 32–84 mmHg] in ten non-responders [seven OP, one DAD, two DILI]). There was no significant difference in the cumulative prednisolone dose between responders and non-responders (median 500 mg [500–4000 mg] vs 600 mg [500–1180 mg]; *p* = 0.292).

ICU and hospital mortality in the whole population were as high as 35.7 and 40.5%, respectively. Patients with a histological diagnosis of OP showed a trend towards lower ICU mortality (21.7% vs 50.0%; *p* = 0.079) and lower hospital mortality (26.1% vs 57.1%; *p* = 0.062). In contrast, there was no difference in ICU mortality (29.4% vs 35.0%; *p* = 0.721) or hospital mortality (29.4% vs 45.0%; *p* = 0.337) of patients with and without a change in therapy due to the histopathological result of the TBLB.

**Fig. 1:**
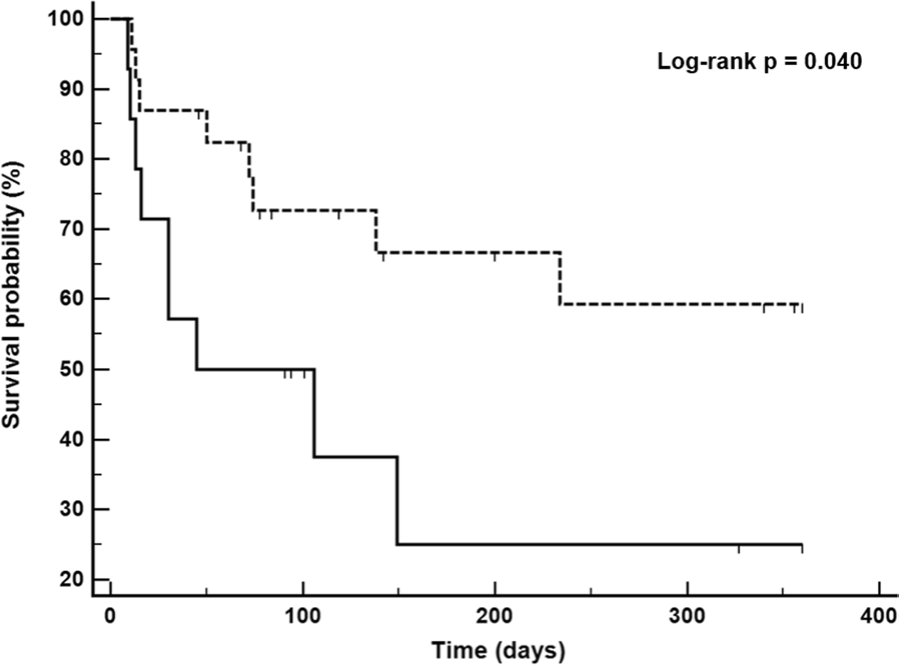
Kaplan-Meier survival analysis – comparison of patients with and without histologically confirmed organizing pneumonia. Legend. Kaplan-Meier survival analysis comparing patients with (*dashed line*) and without (*continuous line*) histological diagnosis of organizing pneumonia

In the multivariable binary logistic regression analysis, age (odds ratio 1.070, 95%CI 1.006–1.138; *p* = 0.031) and the presence of a histological pattern of OP (0.182, [0.036–0.926]; *p* = 0.040) were independently associated with hospital mortality (see Additional file 1: Table S4). This benefit in terms of survival in patients with OP was confirmed by Kaplan-Meier analysis (Fig. 1).

There was no independent association of AE with coagulation parameters, the presence of anticoagulants, or with ventilator variables and respiratory indices (e.g. PEEP, IPAP or mechanical power), and no variable could reliably predict a histological diagnosis of OP before TBLB or a response to glucocorticoid treatment.

## Discussion

To the best of our knowledge, the present study examined the largest number of critically ill patients (without prior lung transplantation) who underwent a transbronchial lung biopsy due to unexplained pulmonary infiltrates while receiving mechanical ventilation, and the results can be summarized as follows. Diagnostic yield of TBLB in this group of patients was high (88%). Serious, life-threatening complications were rare events, but they can’t be completely excluded. In contrast, the impact of the histological result on change in therapy of those patients was low and had no perceptible effect on patient’s outcome. For patients with histologically confirmed organizing pneumonia, there was evidence of improved survival.

Diagnostic yield of TBLB was high when compared to previous studies. O’Brien and colleagues reported a diagnostic yield of 34.9% in 71 patients with in total 83 TBLB procedures [6]. The proportion of patients with prior lung transplantation or immunosuppression was higher (72%) than in the present study and only 2.5% of all patients showed a specific pathology of OP. In contrast, Bulpa et al. demonstrated a yield of 63%, which increased to a maximum of 74% by combination with BAL [7]. Again, the proportion of histologically confirmed OP was low (10.5%), which may explain the relatively higher diagnostic yield in the present study [15]. Another study on mechanically ventilated, lung transplant recipients revealed predominantly pathologies of acute rejection, often combined with DAD. Diagnostic yield of TBLB was 56.4% and the gain in histological diagnoses by SLB was up to 33%, which was associated with a change in therapy in 37% [8]. This was predominantly due to low sensitivity of the TBLB to acute and especially chronic rejection (36 and 0%, respectively). In summary, comparisons of existing studies in terms of diagnostic yield are difficult to interpret due to different patient characteristics (associated with different histological diagnoses) and lack of comparison of TBLB with a reference test (e.g. SLB or autopsy) in most cases.

Complication rate of TBLB was low (11.9%) in the present study, although there was one serious adverse event with ultimately fatal outcome. In contrast, adverse events after SLB in mechanically ventilated patients occur in about 29%, which are mainly attributable to a high number of persistent air-leaks [2]. Pneumothorax incidence (12.5%) was comparable to previous studies [6, 7], although one pneumothorax was related to transbronchial cryobiopsy, and there was no case of persistent air-leak. Incidence of pneumothorax in spontaneously breathing patients varies between 1 and 6% [16, 17], which seems to be slightly lower than in patients receiving mechanical ventilation [6, 7] or for transbronchial cryobiopsy [18]. There is evidence for an increased risk of pneumothorax in spontaneously breathing patients after biopsy of the upper lobes, most likely as a result of the apicobasal pleural pressure gradient in the upright body position (with the most negative pressures in the apex of the thoracic cavity) [17], but there is uncertainty about whether this is also true for mechanically ventilated patients. In addition, the subgroup of ventilated lung transplant recipients probably experience an even lower pneumothorax risk due to pre-existing pleural adhesions [9]. No serious bleeding events occurred, which previously have been reported in up to 5% for transbronchial forceps biopsy [19]. The low AE rate in the present study may be at least in part attributed to the experience of the bronchoscopists, probably preventing the broad applicability of this technique in the ICU. In summary, the more favorable risk profile of the TBLB compared to the SLB must be weighed against its lower diagnostic yield, leading to a higher probability of misclassification of a disease, which can be fatal in the case of respiratory failure with mechanical ventilation, since an incorrect treatment is ineffective or may be even harmful.

Therapeutic consequence of TBLB was low in the present study, with most frequently corticosteroid treatment started. Response to steroids largely depends on the histological diagnosis and is high in patients with eosinophilic pneumonia or COP, although response in patients with SOP is often unpredictable [20]. Responsiveness was defined analogously to previous studies based on changes in the P/F ratio after 7 days of corticosteroid therapy [12, 13]. This may insufficiently reflect the course of the disease, since pulmonary infiltrates often regress over several weeks and assessment of radiographic changes over time was not part of our analyses.

In-hospital mortality was high in the present study and this is in line with a meta-analysis on SLB performed in mechanically ventilated patients, showing a hospital mortality of up to 60% depending on the histological diagnosis [2], which is much higher compared to patients subjected to an elective SLB for suspected interstitial lung disease (ranging between 1.7–6.4%) [21, 22]. Patients with a histopathology of OP showed a favorable clinical outcome, which may in part be attributable to the frequently observed change in therapy (e.g. initiation of corticosteroid treatment), but also may be related to an occasionally observed spontaneous improvement of the disease, which has been reported for COP [23]. Gerard and co-workers showed similar results, demonstrating improved survival of ARDS patients with a steroid-sensitive pathology on SLB, including OP in 21.6% [3]. In contrast, patients with a change in therapy based on the histopathological result showed no advantage in survival in the present study, which may be either related to the small number of cases, but fundamentally raises the question of the benefit of the TBLB in such patients.

Our study has several limitations. First, it was a retrospective, monocentric analysis, and the number of patients assessed was small. Therefore, external validity is uncertain and interpretation of the results should be done with caution. Moreover, the statistical power to precisely define the impact of treatment changes on patient prognosis is limited, and a larger trial (e.g. with a greater proportion of immunosuppressed patients) may lead to different results. Another major limitation is the lack of comparison of the histological results of the TBLB with those of a reference test, i.e. SLB as the gold standard. Diagnostic accuracy of the TBLB, which reflects the percentage of histologically true positive (test positive and diseases present) and true negative (test negative and disease absent) classified patients, can only be determined with such a comparison. So far, this only exists for mechanically ventilated lung transplant recipients [8], and for patients with interstitial lung disease subjected to transbronchial cryobiopsy [24]. No patient subsequently underwent SLB in the present study, most probably due to the high diagnostic yield of TBLB, and the low percentage (16%) of immunosuppressed patients (limiting differential diagnoses of the pulmonary infiltrates). In this context, the additional diagnostic benefit of the SLB may have appeared to be low, leading to reluctance in performing this procedure, considering its significant morbidity and mortality.

## Conclusions

Diagnostic yield of transbronchial lung biopsy in mechanically ventilated, critically ill patients with unexplained pulmonary infiltrates was high and therefore appears to be a suitable alternative to SLB. Complication rate is low, although serious and fatal adverse events can’t be completely excluded, which must be weighed against the limited therapeutic consequence and the apparent lack of benefit in survival in patients with a change in treatment based on the histopathological result. Further prospective studies are required to evaluate the true impact of histopathology on treatment decisions and patient outcomes, and to compare forceps biopsy with cryobiopsy.

## Supplementary Information

**Additional file 1: Table S1**. Comparison of CT features and CT diagnoses with the histopathological diagnoses obtained from transbronchial lung biopsy – all patients (*n* = 42). **Table S2**. Description of the histopathological patterns obtained from transbronchial lung biopsy – all patients (n = 42). **Table S3**. Comparison of broncho-alveolar lavage (BAL) with the histopathological diagnoses obtained from transbronchial lung biopsy (*n* = 21). **Table S4**. Results of the multivariable binary logistic regression analysis.

## Data Availability

The datasets used and/or analyzed during the current study are available from the corresponding author on reasonable request.

## Abbreviation

95%CI: 95% confidence interval
AE: Adverse event(s)
aPTT: activated partial thromboplastin time
ARDS: Acute respiratory distress syndrome
BAL: Broncho-alveolar lavage
BL: Bronchial lavage
COP: Cryptogenic organizing pneumonia
DAD: Diffuse alveolar damage
DILI: Drug-induced lung injury
ICU: Intensive care unit
FiO_2_: Fraction of inspired oxygen
ILD: Interstitial lung disease
INR: International Normalized Ratio
LOS: Length of stay
OP: Organizing pneumonia
P/F ratio: Ratio of partial pressure of oxygen to fraction of inspired oxygen
RLL: Right lower lobe
RUL: Right upper lobe
SLB: Surgical lung biopsy
SOP: Secondary organizing pneumonia
TBLB: Transbronchial lung biopsy
