# Supplementary material for: Diagnostic yield, safety, and impact of transbronchial lung biopsy in mechanically ventilated, critically ill patients: a retrospective study
Source: BMC Pulm Med. 2021 Jan 7;21:15. doi: 10.1186/s12890-020-01357-7 (PMC7788549; doi:10.1186/s12890-020-01357-7)
Supplement: Supplementary file 1 — Additional file 1: Table S1. Comparison of CT features and CT diagnoses with the histopathological diagnoses obtained from transbronchial lung biopsy – all patients (n = 42). Table S2. Description of the histopathological patterns obtained from transbronchial lung biopsy – all patients (n = 42). Table S3. Comparison of broncho-alveolar lavage (BAL) with the histopathological diagnoses obtained from transbronchial lung biopsy (n = 21). Table S4. Results of the multivariable binary logistic regression analysis. [file 12890_2020_1357_MOESM1_ESM.pdf]

Diagnostic yield, safety, and impact of transbronchial lung biopsy in mechanically ventilated, critically ill patients: a retrospective study

Alessandro Ghiani, MD and Claus Neurohr, MD

Additional file 1

1. **Table S1 (Page 2)**
2. **Table S2 (Page 3)**
3. **Table S3 (Page 4)**
4. **Statistical analysis – Table S4 (Page 5)**
5. **Abbreviation list (Page 6)**
6. **References (Page 7)**

Table S1: Comparison of CT features and CT diagnoses with the histopathological diagnoses obtained from transbronchial lung biopsy – all patients (n = 42)

Patient ID	Consolidation	Pleural effusion	GGO	Lymph node enlargement	Atelectasis	Emphysema	Nodules	Bronchiectasis	Cavitary lesion	Pneumothorax*	Reticular pattern	Air trapping	CT diagnoses	Histological diagnoses
**1**	1	1	0	0	0	1	0	0	0	1	0	0	n.s.	**OP**
**2**	1	1	0	1	0	0	0	0	0	0	0	0	n.s.	**OP**
**3**	1	1	0	1	0	0	1	0	0	0	0	0	**sarcoidosis, silicosis**	**silicosis**
**4**	1	1	1	0	0	0	0	0	0	0	0	0	n.s.	**OP**
**5**	1	1	0	0	1	0	0	0	0	0	0	0	n.s.	**OP**
**6**	1	1	1	0	0	0	0	0	0	0	0	0	n.s.	**OP**
**7**	1	1	1	0	0	0	0	0	0	0	0	0	n.s.	**DAD**
**8**	0	0	1	0	0	0	0	0	0	0	0	0	n.s.	**AE-ILD**
**9**	1	0	1	1	0	0	0	0	0	0	0	0	n.s.	**OP**
**10**	1	1	0	0	1	0	0	0	0	0	0	0	n.s.	*non diagnostic*
**11**	0	1	1	0	0	0	0	0	0	0	0	0	**COP**	**OP**
**12**	1	1	0	0	0	0	0	0	0	0	0	0	n.s.	**DILI**
**13**	1	1	1	0	0	0	0	0	0	0	0	0	n.s.	**DAD**
**14**	1	1	0	0	0	1	0	0	0	0	0	0	n.s.	**OP**
**15**	0	0	1	0	0	0	0	0	0	0	0	0	n.s.	**DAD**
**16**	1	0	0	1	0	0	1	0	0	0	0	1	**HP, sarcoidosis**	**Tb**
**17**	1	1	0	0	1	1	0	0	1	1	0	0	n.s.	**OP**
**18**	1	1	0	1	1	0	0	0	0	0	0	0	n.s.	**OP**
**19**	1	1	1	1	1	0	0	0	0	0	0	0	n.s.	**OP**
**20**	1	1	0	0	1	0	0	0	0	0	0	0	n.s.	**pneumonia**
**21**[$]	1	0	1	0	0	0	0	0	0	0	1	0	n.s.	**DILI**
**22**	0	0	1	1	0	0	0	0	0	0	0	0	n.s.	**OP**
**23**[$]	1	1	0	1	0	0	1	0	0	0	0	0	n.s.	*non diagnostic*
**24**[$]	1	1	0	1	0	0	0	0	0	0	0	0	n.s.	**OP**
**25**	1	0	0	1	0	0	0	1	0	0	0	0	n.s.	**DILI**
**26**	1	1	1	0	0	0	1	0	1	0	0	0	n.s.	**NSCLC**
**27**	1	1	0	0	0	0	0	0	0	0	0	0	n.s.	**pneumonia**
**28**	1	1	0	0	0	1	0	0	0	0	0	0	n.s.	*non diagnostic*
**29**	1	0	0	1	0	0	0	0	0	0	0	0	n.s.	**OP**
**30**	1	0	0	1	0	0	0	0	0	0	0	0	n.s.	**OP**
**31**	1	1	0	0	0	0	0	0	0	0	0	0	n.s.	**OP**
**32**	0	1	1	1	0	0	0	0	0	0	0	0	n.s.	*non diagnostic*
**33**	1	0	0	0	0	0	0	0	0	0	0	0	n.s.	**OP**
**34**	0	0	1	0	0	0	0	1	0	0	1	0	**AIP**	**DAD**
**35**	0	1	1	1	0	1	0	0	0	0	0	0	n.s.	**OP**
**36**	1	0	0	1	0	0	0	1	0	1	0	0	n.s.	**OP**
**37**	1	1	0	0	0	0	0	0	0	0	0	0	n.s.	**DILI**
**38**	1	1	0	0	0	1	0	0	0	0	0	0	n.s.	*non diagnostic*
**39**	1	1	0	1	0	0	0	0	0	0	0	0	n.s.	**OP**
**40**	1	1	0	0	0	0	0	0	0	0	0	0	n.s.	**OP**
**41**	1	1	0	0	0	0	0	0	1	0	0	0	**Tb**	**OP**
**42**	1	0	1	0	0	0	0	1	0	0	0	0	**AIP**	**OP**
**summary**	35	29	16	16	6	6	4	4	3	3	2	1		

Legend

Abbreviations: GGO, ground glass opacity; CT, computed tomography; n.s., non specific; OP, organizing pneumonia; DAD, diffuse alveolar damage; AE-ILD, acute exacerbated interstitial lung disease; COP, cryptogenic organizing pneumonia; DILI, drug induced lung injury; HP, hypersensitivity pneumonitis; Tb, tuberculosis; NSCLC, non-small cell lung cancer; AIP, acute interstitial pneumonia

*: Residual pneumothorax despite prior chest tube insertion

[$]: Patients subjected to transbronchial cryobiopsy

Table S2: Description of the histopathological patterns obtained from transbronchial lung biopsy – all patients (n = 42)

**Patient ID**	**Intra-alveolar (myo)fibroblasts**	**AAM**	**AAEC**	**Alveolar hemorrhage***	**Alveolar septal fibrosis**	**Alveolar septal edema**	**IAFD**	**Multinucleated giant cells**	**Hyaline membranes**	**Silica / anthracotic pigments**	**Epithelioid cell granuloma**	**Perivascular histiocytes**	**Carcinoma**	**Histological diagnoses**
**1**	1	1	1	0	1	0	0	0	0	0	0	0	0	**OP**
**2**	1	1	0	0	0	0	0	0	0	0	0	0	0	**OP**
**3**	0	0	0	0	0	0	0	0	0	1	0	1	0	**silicosis**
**4**	1	1	1	0	0	0	0	0	0	0	0	0	0	**OP**
**5**	1	1	0	1	1	1	0	0	0	1	0	0	0	**OP**
**6**	1	1	1	0	0	0	1	0	0	0	0	0	0	**OP**
**7**	0	0	1	1	0	0	1	0	0	0	0	0	0	**DAD**
**8**	0	0	1	0	1	1	0	1	0	0	0	0	0	**AE-ILD**
**9**	1	1	0	0	0	0	0	0	0	0	0	0	0	**OP**
**10**														*non diagnostic*
**11**	1	0	1	0	1	0	0	0	0	0	0	0	0	**OP**
**12**	1	1	0	1	0	0	0	0	1	0	0	0	0	**DILI**
**13**	0	0	0	1	0	0	1	0	0	0	0	0	0	**DAD**
**14**	1	0	0	0	0	0	0	0	0	0	0	0	0	**OP**
**15**	1	1	0	0	0	1	0	0	1	0	0	0	0	**DAD**
**16**	0	0	0	0	0	0	0	1	0	0	1	0	0	**Tb**
**17**	1	1	0	0	0	0	0	0	0	0	0	0	0	**OP**
**18**	1	1	0	0	0	1	0	0	0	0	0	0	0	**OP**
**19**	1	0	1	0	0	0	0	0	0	0	0	0	0	**OP**
**20**	0	1	0	1	0	0	0	0	0	0	0	0	0	**pneumonia**
**21**[$]	1	0	0	0	0	0	0	0	0	0	0	0	0	**DILI**
**22**	1	1	0	0	0	0	0	0	0	0	0	0	0	**OP**
**23**[$]														*non diagnostic*
**24**[$]	1	1	0	0	0	0	0	0	0	0	0	0	0	**OP**
**25**	1	1	1	0	0	0	0	0	0	0	0	0	0	**DILI**
**26**	0	0	0	0	0	0	0	0	0	0	0	0	1	**NSCLC**
**27**	0	0	0	0	0	0	0	0	0	0	0	0	0	**pneumonia**
**28**														*non diagnostic*
**29**	0	1	0	1	0	0	0	1	0	0	0	0	0	**OP**
**30**	1	1	0	0	0	0	0	0	0	0	0	0	0	**OP**
**31**	1	0	0	1	0	0	0	0	0	0	0	0	0	**OP**
**32**														*non diagnostic*
**33**	1	1	0	0	0	0	0	0	0	0	0	0	0	**OP**
**34**	0	1	1	0	0	0	1	0	0	0	0	0	0	**DAD**
**35**	1	0	1	0	0	0	0	0	0	0	0	0	0	**OP**
**36**	1	1	0	0	0	0	0	0	0	0	0	0	0	**OP**
**37**	0	1	1	0	1	1	1	0	0	0	0	0	0	**DILI**
**38**														*non diagnostic*
**39**	0	1	0	0	0	0	0	0	0	0	0	0	0	**OP**
**40**	1	1	1	0	0	0	0	0	0	0	0	0	0	**OP**
**41**	1	0	1	0	1	0	0	0	0	0	0	0	0	**OP**
**42**	1	0	0	0	0	0	0	0	0	0	0	0	0	**OP**
**summary**	25	22	13	7	6	5	5	3	2	2	1	1	1	

Legend

Abbreviations: AAM, activated alveolar macrophages; AAEC, activated alveolar epithelial cells; IAFD, intra-alveolar fibrin deposition; OP, organizing pneumonia; DAD, diffuse alveolar damage; AE-ILD, acute exacerbated interstitial lung disease; DILI, drug induced lung injury; Tb, tuberculosis; NSCLC, non-small cell lung cancer

*: Classified as artifacts associated with the transbronchial forceps biopsy

[$]: Patients subjected to transbronchial cryobiopsy

Table S3: Comparison of broncho-alveolar lavage (BAL) with the histopathological diagnoses obtained from transbronchial lung biopsy (n = 21)

**Patient ID**	**Target lung lobe for BAL**	**BAL recovery (mL)**	**Total cell count ($*10^6$/100 mL)**	**Neutrophils (%)**	**Eosinophils (%)**	**Lymphocytes (%)**	**Alveolar macrophages (%)**	**Histological diagnoses**
**2**	ML	33	3.3	34	0	4	62	**OP**
**4**	ML	30	15	90	0	0	10	**OP**
**6**	ML	41	8.2	12	0	20	68	**OP**
**13**	ML	33	3.3	25	1	1	73	**DAD**
**14**	ML	23	4.6	15	7	19	59	**OP**
**15**	ML	40	16	3	0	23	73	**DAD**
**16**	LB4/5	21	2.1	16	2	28	54	**Tb**
**18**	ML	42	8.4	3	1	3	93	**OP**
**19**	RUL	50	25	85	0	0	15	**OP**
**21**[$]	ML	50	10	60	2	10	28	**DILI**
**23**[$]	ML	30	3	21	0	8	71	*non diagnostic*
**24**[$]	LB4/5	45	4.5	40	12	5	43	**OP**
**25**	ML	34	34	72	0	8	20	**DILI**
**26**	ML	50	25	22	2	2	74	**NSCLC**
**28**	LB4/5	23	4.6	70	0	0	30	*non diagnostic*
**30**	LB4/5	20	2	80	1	4	15	**OP**
**31**	RUL	35	17.5	77	1	1	21	**OP**
**37**	RLL	12	3.6	45	1	7	47	**DILI**
**39**	LB4/5	26	15.6	95	0	0	5	**OP**
**40**	ML	34	13.6	17	1	4	79	**OP**
**42**	LB4/5	26	10	59	1	12	28	**OP**

Legend

Normal cell distribution of BAL refers to ≥ 85% of alveolar macrophages, ≤ 15% lymphocytes, ≤ 3% neutrophils, and ≤ 1% eosinophils.

Abbreviations: BAL, broncho-alveolar lavage; ML, middle lung lobe; OP, organizing pneumonia; DAD, diffuse alveolar damage; LB4/5, lingula lung segments; Tb, tuberculosis; RUL, right upper lobe; DILI, drug induced lung injury; NSCLC, non-small cell lung cancer; RLL, right lower lobe

[$]: Patients subjected to transbronchial cryobiopsy

Statistical analysis

The results from the multivariable binary logistic regression analysis (using forward selection, entry criteria p = 0.05 and stay criteria p = 0.2) can be summarized as follows: In terms of hospital mortality, only the variable *age* and the presence of histologically confirmed *organizing pneumonia* (OP) were independent risk factors and entered the final model [Table S4].

Table S4: Results of univariate und multivariable binary logistic regression analysis [**1**]

**Univariable regression model**				
**log-odds***	L = – 4.193 + (0.056 x age)			
**OR (95%CI); p value**	**age**	1.058 (1.001 – 1.119); p = 0.047		
**AUROC (95%CI)**	0.70 (0.54 – 0.83)			
**2 x 2 confusion matrix**	**TP** 9		**FP** 8	
	**FN** 7		**TN** 18	
**Diagnostic metrics derived from 2 x 2 table (95%CI)**	**Sensitivity**	56% (30-80)	**PLR**	1.83
	**Specificity**	69% (48-86)	**NLR**	0.63
	**PPV**	53% (35-70)	**DOR**	2.9
	**NPV**	72% (58-83)	**F1 score**	0.54
	**Accuracy**	64% (48-78)	**MCC**	0.25
**Multivariable regression model**				
**log-odds***	L = – 4.061 + (0.068 x age) – (1.706 x OP)			
**OR (95%CI); p value**	**age**	1.070 (1.006 – 1.138); p = 0.031		
	**OP**	0.182 (0.036 – 0.926); p = 0.040		
**AUROC (95%CI)**	0.81 (0.66 – 0.92)			
**2 x 2 confusion matrix**	**TP** 9		**FP** 8	
	**FN** 7		**TN** 18	
**Diagnostic metrics derived from 2 x 2 table (95%CI)**	**Sensitivity**	50% (23-77)	**PLR**	3.83
	**Specificity**	87% (66-97)	**NLR**	0.58
	**PPV**	70% (42-88)	**DOR**	6.6
	**NPV**	74% (62-83)	**F1 score**	0.58
	**Accuracy**	73% (56-86)	**MCC**	0.40

Legend

Abbreviations: L, log-odds; OP, organizing pneumonia; OR, odds ratio; 95%CI, 95% confidence interval; AUROC, area under the receiver operating characteristic curve; TP, true positive; FP, false positive; FN, false negative; TN, true negative; PPV, positive predictive value; NPV, negative predictive value; PLR, positive likelihood ratio; NLR, negative likelihood ratio; DOR, diagnostic odds ratio; MCC, Matthews correlation coefficient [**2**]

*****: The following candidate predictors did not enter the model: (Male) gender, APACHE-II, Charlson comorbidity index, ventilator days on transbronchial lung biopsy (TBLB), vasopressors, therapeutic consequence from TBLB, coronary artery disease, extracorporeal lung assistance, immunosuppression, and malignancy

Abbreviation list

- 95%CI: 95% confidence interval
- AAEC: Activated alveolar epithelial cells
- AAM: Activated alveolar macrophages
- AE-ILD: Acute exacerbated interstitial lung disease
- AIP: Acute interstitial pneumonia
- AUROC: Area under the receiver operating characteristic curve
- BAL: Broncho-alveolar lavage
- COP: Cryptogenic organizing pneumonia
- CT: Computed tomography
- DAD: Diffuse alveolar damage
- DILI: Drug induced lung injury
- DOR: Diagnostic odds ratio
- FN: False negative
- FP: False positive
- GGO: Ground glass opacity
- HP: Hypersensitivity pneumonitis
- IAFD: Intra-alveolar fibrin deposition
- n.s.: non specific
- L: Log-odds
- LB4/5: Lingula lung segments
- MCC: Matthews correlation coefficient
- ML: Middle lung lobe
- NLR: Negative likelihood ratio
- NPV: Negative predictive value

- NSCLC: Non-small cell lung cancer
- OP: Organizing pneumonia
- OR: Odds ratio
- PLR: Positive likelihood ratio
- PPV: Positive predictive value
- RLL: Right lower lobe
- RUL: Right upper lobe
- Tb: Tuberculosis
- TN: True negative
- TP: True positive

References

1. Leisman DE, Harhay MO, Lederer DJ, et al.: Development and reporting of prediction models: Guidance for authors from editors of respiratory, sleep, and critical care journals. *Crit Care Med* 2020;48:623–33
2. Chicco D, Jurman G: The advantages of the Matthews correlation coefficient (MCC) over F1 score and accuracy in binary classification evaluation. *BMC Genomics* 2020;21:6
